# Outcomes following liver trauma in equestrian accidents

**DOI:** 10.1186/1752-2897-8-13

**Published:** 2014-08-21

**Authors:** Anita Balakrishnan, Reyad Abbadi, Kathryn Oakland, Saurabh Jamdar, Simon JF Harper, Neville V Jamieson, Emmanual L Huguet, Asif Jah, Raaj K Praseedom

**Affiliations:** 1 Department of Hepatopancreaticobiliary Surgery, Addenbrooke’s Hospital and Cambridge University, Hills Road, Cambridge CB2 0QQ, UK; 2 Department of Hepatopancreaticobiliary Surgery, Bristol Royal Infirmary, Marlborough Street, Bristol BS2 8HW, UK; 3 Hepatobiliary Surgery Unit, Manchester Royal Infirmary, Oxford Road, Manchester M13 9WL, UK

**Keywords:** Liver trauma, Equine accidents, Liver injury

## Abstract

**Background:**

Equestrian sports are common outdoor activities that may carry a risk of liver injury. Due to the relative infrequency of equestrian accidents the injury patterns and outcomes associated with liver trauma in these patients have not been well characterized.

**Methods:**

We examined our experience of the management of equestrian liver trauma in our regional hepatopancreaticobiliary unit at a tertiary referral center. The medical records of patients who sustained liver trauma secondary to equestrian activities were analysed for parameters such as demographic data, liver function tests, patterns of injury, radiological findings, the need for intervention and outcomes.

**Results:**

20 patients sustained liver trauma after falling from or being kicked by a horse. The majority of patients were haemodynamically stable on admission. Alanine transaminase (ALT) levels were elevated in all patients and right-sided rib fractures were a frequently associated finding. CT demonstrated laceration of the liver in 12 patients, contusion in 3 and subcapsular haematoma in 2. The right lobe of the liver was most commonly affected. Only two patients required laparotomy and liver resection; the remaining 18 were successfully managed conservatively.

**Conclusions:**

The risk of liver injury following a horse kick or falling off a horse should not be overlooked. Early CT imaging is advised in these patients, particularly in the presence of high ALT levels and concomitant chest injuries such as rib fractures. Despite significant liver trauma, conservative management in the form of close observation, ideally in a high-dependency setting, is often sufficient. Laparotomy is only rarely warranted and associated with a significantly higher risk of post-operative bile leaks.

## Introduction

Horse-riding accidents are common with over 100,000 cases in the USA annually [1]. Smaller numbers occur in the UK at around 260 per year yet still constitute 10% of all sports injuries [2]. The liver is the second most frequently injured intra-abdominal organ after the spleen in cases of blunt abdominal trauma, despite its relatively well-protected location under the costal margin [3,4]. Horses can weigh up to 1100 pounds and are capable of reaching speeds up to 40mph. In addition a kick from a horses’ hoof has been shown to deliver over 10,000 Newtons of force to its victim or 1.8 times its body weight [5,6]. It is not surprising therefore that although liver injuries constituted less than 1% of all equestrian injuries in several studies in the literature these injuries can be severe and carry life-threatening consequences for the patients involved [7,8]. The morbidity and mortality resulting from liver trauma are often a product of associated chest and other intra-abdominal injuries as well as complications such as haemorrhage and sepsis [4,9]. Mortality rates following blunt hepatic trauma have decreased to below 10% in the past several decades however despite this the management of hepatic trauma remains a difficult challenge [10].

Equine-associated liver injuries are not well studied due to the relative infrequency of these patients at most hospitals. Our tertiary hepatopancreatobiliary (HPB) center is in close proximity to a major racecourse, providing us an excellent opportunity to examine our experience of equine-associated injuries to the liver. Our data show that a high index of suspicion must be maintained for liver injuries following equine-related injuries with early cross-sectional imaging to identify the extent of injury. These patients can predominantly be managed non-operatively but require close observation in a high-dependency setting to detect any deterioration or complications.

## Methods

We examined our experience of the management of equine-associated liver trauma in our regional hepatopancreaticobiliary unit at a tertiary referral center. Patients who had sustained liver injuries between January 1995 and December 2011 were identified via keyword searches of clinical coding records and the diagnosis confirmed via discharge summaries, radiological reports and clinic letters.

The medical records of patients who sustained liver trauma secondary to equestrian activities during this time period were interrogated for parameters such as demographic data such as age and gender, haematological and biochemical parameters including haemoglobin counts and liver function tests. Ultrasound (USS) and computed tomography (CT) reports, and images where available, were reviewed to identify specific patterns of injury associated with liver trauma in this patient group. The severity of liver injury was estimated according to the Abbreviated Injury Scale (AIS) based on review of notes and radiological imaging where available. Overall injury severity was calculated according to the Injury Severity Score (ISS). Any surgical or radiological intervention performed on these patients was specifically noted. In addition, management outcomes in the form of length of stay and survival were examined.

Data are presented as medians and ranges unless stated otherwise. Differences between groups were analysed for statistical significance using Fisher’s exact test.

## Results

### Demographic data

263 patients sustained liver trauma in the 15-year period between January 1995 and December 2011, necessitating transfer to our regional unit. In the majority of cases this was due to motor vehicle accidents. 20 of these patients (7.6%) sustained liver trauma after falling from or being kicked by a horse; this is the group of patients that will be examined in this study.

The median age of the patients who sustained liver trauma from an equine-related injury was 22 years (range 5–48). 2 patients were male and the remaining 18 were female. Four patients had a history of asthma and one had previous breast cancer, the remaining patients had no significant comorbidities. The majority of patients were clinically stable on admission; only three patients exhibited signs of haemodynamic compromise in the form of tachycardia and hypotension.

### Blood results

Routine haematological and biochemical parameters were examined in all patients upon arrival to the Accident and Emergency Department (Table 1). Of the liver function tests examined, alanine transaminase (ALT) levels were elevated in all patients with a median level of 72 IU/L (range 60–925 IU/L). Bilirubin levels were normal in all but one patient (median 7μmols/L, range 2-41 μmols/L). Similarly alkaline phosphatase (ALP) levels were only elevated in 2 patients with an overall median level of 72 IU/L (range 35–183 IU/L). The majority of patients had normal haemoglobin measurements (median 12 g/dl, range 9.7-16 g/dl); this was below the normal range of 11-15 g/dl in only 4 patients.

**Table 1:** Biochemical and haematological parameters

**Blood test**	**Median**	**Range**	**Normal range**
Bilirubin (μmols/L)	7	2-41	2 - 17
Alanine transaminase (ALT; IU/L)	72	60-925	10 - 40
Alkaline phosphatase (ALP; IU/L)	229	35-183	25 - 135
Haemoglobin (Hb; g/dL)	12	9.7-16	11-15

### Imaging findings

**Figure 1:**
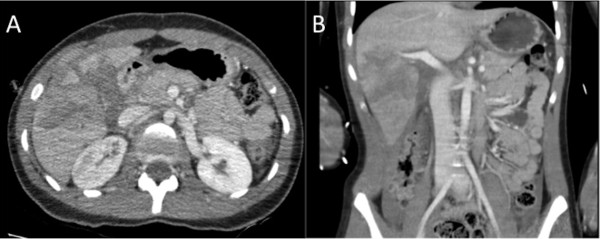
**CT of the abdomen with contrast of hepatic injury following a kick by a horse.** Axial **(A)** and coronal **(B)** images show lacerations involving the right lobe of the liver, which settled with conservative management.

Imaging of the abdomen in the form of either USS or CT was performed in the 19 of the 20 patients (Table 2). One patient was found to be too unstable on admission for imaging and hence was taken straight to theatre. Abdominal USS was performed in 9 of the 20 patients, revealing free fluid in four patients and subcapsular haematoma in the remaining 5. The commonest site of free fluid in these patients was in the subhepatic space. 7 patients who had had an USS went on to have a CT.CT of the abdomen and pelvis with contrast was performed in 17 patients. 2 patients had ultrasonography as the only form of abdominal imaging. One of these two patients was relatively unstable at the time of admission and was taken to theatre on the basis of the USS confirming a haematoma within the right hemiliver. This patient was too unstable for any form of abdominal imaging and the decision was therefore made to proceed to laparotomy. CT was the only form of abdominal imaging in 10 patients, demonstrating laceration of the liver in 12 patients, contusion in 3 and subcapsular haematoma in 2. Only one patient in our study had signs of contrast extravasation on CT scan. Indications for embolization in our unit include evidence of arterial extravasation on CT and haemodynamic instability or need for ongoing blood transfusions to maintain haemoglobin levels, or as adjunctive haemorrhage control in patients with controlled bleeding despite laparotomy. This patient remained haemodynamically stable throughout hence no embolization was attempted. The right lobe of the liver was most commonly injured (segments V-VIII, Figure 1). Segment IV was injured in 3 patients. None of the patients sustained documented injury to left-sided segments II or III of the liver. None of the patients required primary aortography or venography.

**Table 2:** Imaging findings of hepatic injuries

**Imaging modality (n)**	**Findings**	**Number of patients**
USS (9)	Subcapsular haematoma	5
	Free fluid	4
CT (17)	Laceration	12
	Contusion	3
	Subcapsular haematoma	2

**Figure 2:**
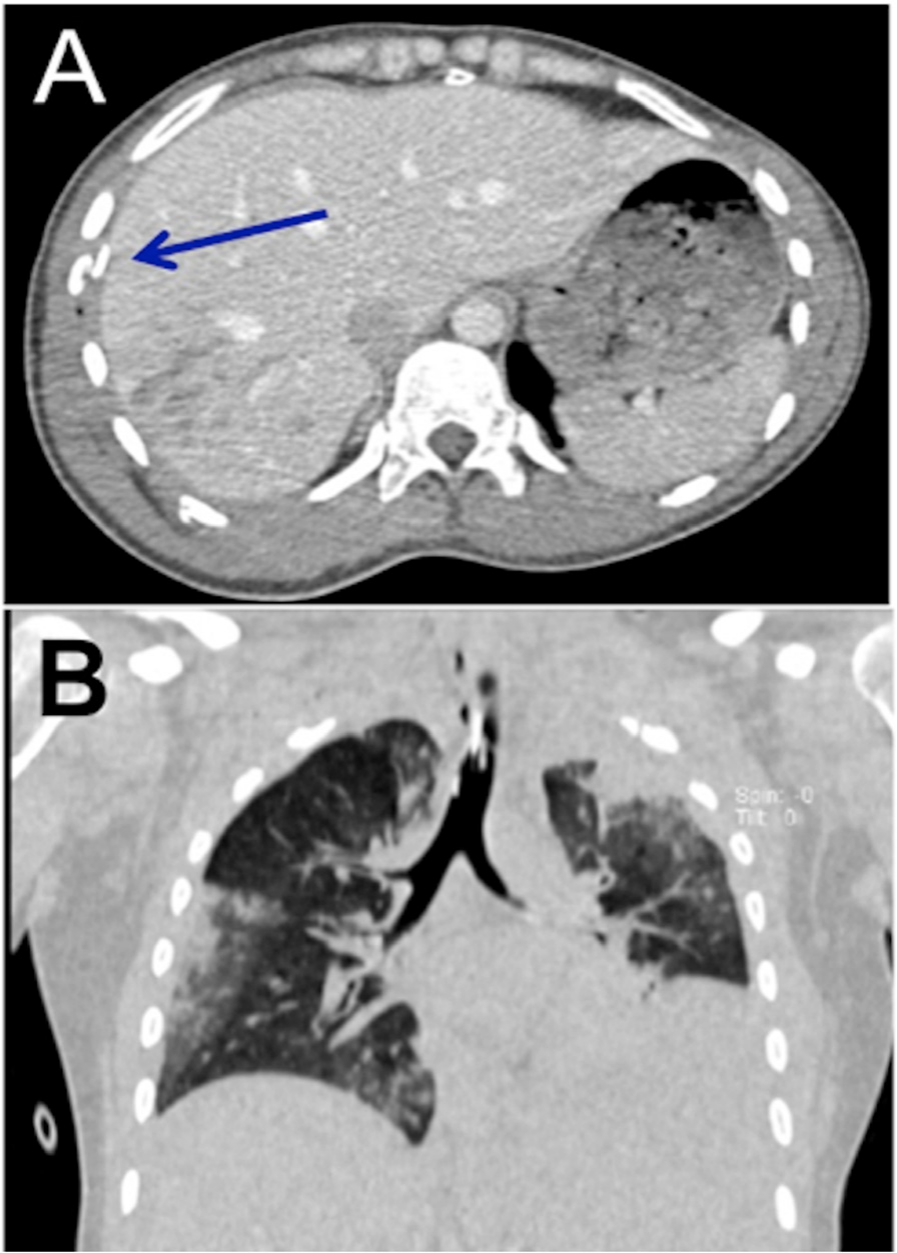
Chest CT in axial cross-section showing right-sided rib fractures (A, arrowed) and coronal section showing lung contusions (B); injuries frequently associated with liver trauma.

Right-sided fractures of the 8^th^ to 11^th^ ribs were the most frequently associated finding often noted on CT of the chest, where performed, or of the chest X-ray as part of the routine trauma series (Figure 2). Other associated injuries were renal lacerations in 4 patients, a cardiac contusion in one patient and pancreatitis in another patient. 2 patients sustained orthopaedic injuries in the form of clavicular and tibiofibular fractures. Hepatic and extra-hepatic injuries sustained by each patient including the Injury Severity Scores are shown in Table 3.

**Table 3:** Hepatic and extra-hepatic injuries sustained by each patient

**Age**	**M/F**	**AIS Grade of liver injury**	**Management**	**Other injuries**	**ISS**
25	F	2	Conservative	Rib fractures	5
20	F	5	Conservative	Cardiac and lung contusions	29
21	F	5	R hemihepatectomy	None	25
5	F	4	Conservative	None	16
48	F	2	Conservative	Rib fractures	5
26	M	2	Conservative	Rib fractures	5
26	F	4	Conservative	Renal laceration	20
17	F	4	Conservative	Renal laceration, rib fractures	22
45	F	2	Conservative	Pancreatic injury	8
25	F	4	Conservative	Rib fractures	17
31	F	4	Conservative	Rib fractures	17
45	F	4	Conservative	Lung contusions	11
20	F	5	Conservative	None	25
18	F	5	R hemihepatectomy	Lower limb fractures	34
17	F	2	Conservative	None	4
22	M	3	Conservative	Rib fractures	10
21	F	3	Conservative	Clavicular and rib fractures, lung contusions	33
42	F	3	Conservative	Rib fractures	18
16	F	4	Conservative	Renal laceration	25
21	F	5	Conservative	Renal laceration	29

### Surgical intervention

**Figure 3:**
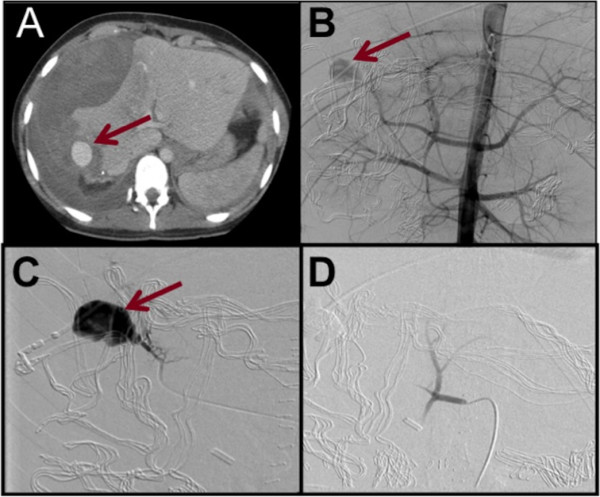
**Right hepatic artery pseudo aneurysm.** Arrows show the aneurysm on axial CT **(A)** and angiography **(B)**. The pseudoaneurysm and feeding vessel **(C)** were coiled and no further filling was demonstrated **(D)**.

Two of the twenty patients who sustained equine-related liver injuries in this time period required laparotomy. Both patients were haemodynamically unstable on admission and in both cases a right hemihepatectomy was performed for control of bleeding. The first patient had haemodynamic instability unresponsive to resuscitation in the context of severe liver trauma (AIS grade 5) and required an emergency laparotomy for haemorrhage control. This patient developed a bile leak in the immediate post-operative period, which settled with conservative management.The second patient underwent emergency right hemihepatectomy at a peripheral hospital and was discharged home 28 days later following conservative management of a bile leak in the immediate post-operative period. This patient was subsequently readmitted as an emergency 3 weeks later with collapse and a right paracolic collection. Re-laparotomy in the original hospital identified massive haemorrhage necessitating packing and transfer to our center where embolization of a right hepatic artery aneurysm was performed 2 days later followed by removal of packs the following day (Figure 3).

One patient had haemodynamic instability but did not require surgical intervention. This patient had not only sustained a liver injury but also lung contusions and bilateral haemothoraces which were managed by intercostal chest drainage. Haemodynamic stability was regained after transfusion of 4 units of packed red cells. No contrast extravasation was noted on CT hence no embolization was performed. The patient required 10 days of intensive care support and suffered no sequelae from the hepatic injury.

The remaining 18 patients were successfully managed non-operatively.

### Short-term and long-term complications

Both patients who were managed operatively sustained bile leaks in the short term, which were managed conservatively, as mentioned above. In contrast, no evidence of bile leak was noted in the patients managed without surgical intervention (p = 0.005). One patient who did not undergo surgical intervention did however develop biliary obstruction at 2 months post-injury due to haemobilia, requiring endoscopic stenting as a temporary measure. The length of stay in our hospital ranged from 3 to 47 days, with a median length of stay of 9 days. None of the patients in this study died in hospital and all were discharged from the outpatient clinic after review. The median length of stay was longer in patients who had surgical intervention compared to those that did not (9 days vs 45 days) and in those patient with other injuries compared to those with isolated liver trauma (11 days vs 9 days). Due to the small number of patients requiring surgical intervention, statistical analysis could not be performed.

## Discussion

Equestrian accidents are a known cause of abdominal injuries yet there is little literature surrounding this. Our study is the largest published series of liver injuries following equestrian accidents and is the first to focus on hepatic injuries following equine-related accidents and the management and outcome of these patients. Our findings suggest that conservative management of these liver injuries is a safe and viable option and highlight the importance of early cross-sectional imaging to aid diagnosis and determine the extent of injury.

The retrospective nature of our study necessarily produces some limitations. Firstly, we cannot comment on the outcome of all equestrian-associated injuries as only liver injuries are referred to our hepatopancreatobiliary center. Secondly, we could not review the imaging from patients admitted more than ten years ago hence we are unable to accurately re-classify the severity of injury (CT grading) in this cohort. The degree of injury and segments of liver involved were hence identified from documentation of radiological reports in the medical notes. Thirdly, as our center is a regional tertiary referral center for hepatopancreatobiliary pathology, the majority of our patients were referred from elsewhere. While we accept that the admission to and initial management in peripheral units introduces an element of heterogeneity into the analysis, this is inevitable given the centralization of hepatopancreatobiliary services in the United Kingdom and is thus a reflection of the reality of liver trauma management in practice. In addition the process of centralization has provided regional units such as ours the benefit of experience of managing more patients with these injuries than would be seen in individual peripheral units.

All patients in our study had abdominal imaging either in the form of an USS or a CT with the exception of one of the patients who required immediate laparotomy for haemodynamic instability as mentioned previously. There was no discrepancy between the CT and USS findings in those patients who had both forms of abdominal imaging however CT was able to provide additional information in the form of identification of contrast extravasation suggestive of active bleeding which could not be detected on USS. Indeed studies of liver trauma from all causes has recommended the use of early CT in preference to USS in the assessment of liver injuries as an adjunct to aiding the decision for conservative management [11]. USS has poorer resolution for detection of solid organ injuries, while CT allows accurate diagnosis of the type and extent of the hepatic injury and detection of haemoperitoneum as well as other associated injuries [12]. While we accept the risk of radiation that CT poses to younger patients in particular, current evidence in the literature as well as Advanced Trauma Life Support® guidelines recommend CT to be better than ultrasound for parenchymal injury, including in the paediatric population [13,14]. In addition CT is less operator dependent than ultrasonography and is especially beneficial in haemodynamically stable patients as unstable patients with evidence of free intraabdominal fluid on Focused Assessment with Sonography in Trauma (FAST) scans are candidates for urgent laparotomy [14]. Our own experience of the value of CT scanning in abdominal pathology has shown that early CT assessment allows the detection of unexpected clinically significant primary and secondary diagnosis, thereby improving patient management [15]. While the diagnostic sensitivity of ultrasound can be augmented with the use of contrast enhancement, ultrasound has still been found insufficient as first line investigation in the trauma patient but may be a useful modality in follow-up of abdominal injuries [16].

Right-sided rib fractures and renal lacerations were commonly associated injuries in our patient group as previously described and serve to highlight the degree of force associated with the mechanism of injury [7]. These were all managed conservatively in our study but such injuries should be observed closely as they can significantly contribute to the overall comorbidity associated with the injury [4].

All but two patients in our study were successfully managed non-operatively. This is in keeping with studies on all-cause liver trauma which demonstrate a trend towards non-operative management as the treatment of choice in haemodynamically stable patients [11,17]. Indeed, anatomical hepatectomies in the setting of liver trauma have been associated with mortality rates approaching 50% in the published literature [9,18]. A safer surgical option in the haemodynamically compromised patient is laparotomy and packing of the liver, which may avoid the need for liver resection and provide a stabilizing measure by tamponading the bleeding until the patient is adequately resuscitated. The aim of non-operative management of liver trauma in the absence of haemodynamic instability is in contrast with the management of enteric injuries, which necessitate urgent laparotomy to minimize peritoneal contamination and sepsis.

None of the patients with equine-related liver injuries who were managed non-operatively required subsequent laparotomy, corroborating other studies showing only a 2.2% failure rate of non-operative management in all-cause liver trauma [11]. Both patients who required laparotomy in our study demonstrated haemodynamic instability despite adequate resuscitation on presentation necessitating immediate surgical intervention for control of bleeding. Both of these patients also subsequently developed bile leaks in contrast to the absence of bile leaks in those managed conservatively. Bile leaks are a known complication of liver trauma with a higher incidence described in the literature following operative management (19%) compared to non-operative management (1.5%) and an overall risk of up to 25% [19,20]. The bile leaks in both patients in our study settled with conservative management, however, if this fails other measures such as laparoscopy or laparotomy with washout and the placement of drains or decompression of the biliary tree via ERCP or PTC may sometimes be indicated [20].

Although the majority of patients are likely to be managed non-operatively, it is vital that these patients are managed in a tertiary center equipped with facilities for adequate monitoring as well as the necessary surgical and radiological expertise should the need for intervention arise. Patients should be observed closely ideally in a high-dependency setting to ensure any signs of haemodynamic instability suggesting clinical deterioration and failure of conservative management are recognized and acted upon early. Radiological intervention such as embolisation is also an increasingly viable alternative in the non-operative management of active bleeding following liver trauma with success rates exceeding 80% [21-24]. This therapeutic modality, however, is not without complications, such as hepatic ischaemia which may lead to hepatic necrosis [25]. Interventional radiological and endoscopic techniques are also a useful treatment modality for delayed complications of liver trauma such as pseudoaneurysms and bile leaks, as demonstrated in our study.

There were no mortalities in our series; overall mortality from liver trauma in the literature is approximately 9% [10] but ranges from 33% to 50% following anatomical resection in the context of injury [11,26].

## Conclusions

The risk of liver injury following a horse kick or falling off a horse should not be overlooked. Early CT imaging is advised in these patients, particularly in the presence of high ALT levels and concomitant chest injuries such as rib fractures. Despite significant liver trauma, usually to the right lobe, laparotomy is rarely warranted and conservative management in the form of close observation in a high-dependency setting is often sufficient.

### Ethical approval

Ethical approval was not required for this study.

## Competing interests

All authors have no non-financial interests that may be relevant to the submitted work.

## Authors’ contributions

AB, RA, NVJ and RKP designed the study. AB, RA and KO collected the data. AB, SJ, SJFH, NVJ, ELH and AJ analysed the data. AB and RKP wrote the paper. All authors read and approved the final manuscript.
